# Effective strategies for cultivating rapid response, team collaboration, and stress resistance in emergency standardized training nurses during real rescue

**DOI:** 10.3389/fmed.2025.1683359

**Published:** 2025-10-06

**Authors:** Ting Feng, Li Hu, Yali Yang, Chan Du, Junjie Li

**Affiliations:** Department of Emergency, Xijing Hospital, The Fourth Military Medical University, Xi'an, Shaanxi, China

**Keywords:** emergency nursing, standardized training, real rescue, rapid response, team collaboration, stress resistance

## Abstract

**Background/purpose:**

Standardized training for emergency nurses should include effective methods to develop rapid response, teamwork, and stress resistance. Traditional simulation-based training may lack the intensity and unpredictability of real emergencies. This study evaluated a progressive real rescue participation strategy compared to traditional simulation training.

**Methods:**

A retrospective analysis included 235 emergency standardized training nurses from May 2022 to April 2025. They were divided into a Traditional Training group (*n* = 126, simulation and theory only) and a Real Rescue Training group (*n* = 109, simulation/theory plus phased participation in actual resuscitation). Validated scales assessed rapid response (TDMI), team climate (TCI), resilience (CD-RISC), burnout (MBI), and critical thinking (CTDI-CV) before and after the 6-month training. Department exit assessments and nursing satisfaction were also compared.

**Results:**

Baseline characteristics were comparable. Both groups improved significantly on all post-training scales. However, the Real Rescue group showed significantly greater improvement than the Traditional group in all dimensions of rapid response (TDMI subscales, all *p* < 0.05), team collaboration (TCI subscales, all *p* < 0.05), stress resistance (CD-RISC subscales, all *p* < 0.05), critical thinking (CTDI-CV subscales, all *p* < 0.05), and reduced burnout (MBI subscales, all *p* < 0.01). The Real Rescue group also scored higher on theoretical (*p* = 0.02) and clinical skills exit assessments (*p* = 0.01) and achieved significantly higher nursing satisfaction (*p* = 0.01).

**Conclusion:**

Real-rescue training is more effective than traditional methods in improving emergency nurses’ critical skills and psychological resilience. This approach should be integrated into standardized training programs to better prepare nurses for real-world emergencies.

## 1 Introduction

Emergency departments are characterized as high-pressure environments due to the critical nature of medical interventions required and the potential for significant consequences on patient outcomes. Patient outcomes critically depend on nurses’ abilities to respond rapidly. Nurses must also collaborate seamlessly within multidisciplinary teams and maintain performance under intense pressure. Standardized training programs for new emergency nurses aim to build these essential competencies (1, 2). Traditionally, these programs have heavily relied on simulation-based training. Simulation-based training offers a safe, controlled environment to practice skills and protocols using manikins and scripted scenarios. It allows for repeated practice, standardized experiences, and immediate feedback without patient risk. These benefits make simulation a cornerstone of modern clinical education (3, 4).

However, despite its advantages, simulation training has inherent limitations in replicating the full complexity and psychological demands of actual emergency resuscitation. The controlled nature of simulations can reduce the psychological stress and cognitive load experienced in real life. Simulations may lack the chaotic elements, true urgency, unpredictable patient responses, simultaneous competing demands, and high-stakes consequences inherent in real resuscitation. Consequently, nurses trained primarily through simulation might experience a significant performance gap when transitioning to real clinical practice. It potentially impacts patient safety and nurse confidence (5, 6).

This gap highlights the necessity for training strategies that effectively connect simulated practice with real-world application. Experiential learning theory emphasizes that deep learning occurs through direct experience and reflection (7). Bandura’s social learning theory further suggests that observing and gradually participating in real performances within a team context is crucial for skill acquisition and confidence building (8). Therefore, carefully structured exposure to actual emergency resuscitation during training holds significant potential. Such exposure could provide the missing elements of genuine stress, authentic team dynamics, and the need for real-time adaptive decision-making under pressure (9).

While the potential benefits of real clinical experience are acknowledged, integrating it systematically into standardized training programs presents challenges. Concerns include patient safety, ethical considerations regarding novice participation, potential for increased stress leading to burnout, and the need for structured progression to ensure learning without overwhelming trainees. A phased approach, moving from observation to assisted participation and finally to defined core tasks under close supervision, could mitigate these risks while maximizing learning (10, 11).

Given the global shortage of emergency nurses, optimizing training strategies is essential for sustaining healthcare systems. This study investigates whether a structured program of progressive participation in real emergency resuscitation, integrated alongside traditional simulation and didactic teaching, is more effective than traditional simulation-based training alone. We specifically evaluated its impact on developing the core competencies of rapid response capability, team collaboration, stress resistance, critical thinking, and overall job readiness among emergency standardized training nurses. This study contributes to the evidence base by comparing real-rescue and traditional training through rigorous, multidimensional assessments.

Rapid response, team collaboration, and stress resistance represent core competencies for emergency nurses. In high-acuity settings such as the emergency department, seconds often determine patient survival; delays in triage or decision-making are associated with increased morbidity and mortality. Equally critical is team collaboration, as resuscitation and trauma care demand seamless role clarity, communication, and coordination breakdowns in teamwork are repeatedly linked to preventable errors and poorer outcomes. Finally, stress resistance is indispensable: emergency nurses are routinely exposed to high-stakes, emotionally charged, and unpredictable events, which, if unmanaged, can precipitate burnout, impaired judgment, and compromised care delivery. Together, these domains underpin not only individual clinical performance but also the collective safety culture of emergency departments.

## 2 Materials and methods

### 2.1 Inclusion and grouping of objects

A retrospective analysis was conducted on 235 nurses who completed standardized training in the Department of Emergency of our hospital from May 2022 to April 2025. Inclusion criteria were: (a) newly hired nurses undergoing emergency standardized training; (b) possession of a nursing practice qualification certificate; (c) completion of pre-job training; and (d) voluntary participation in the standardized training program.

Exclusion criteria included those who could not fully participate in the training due to study trips, extended leave (≥ 2 weeks), or job transfers during the training period.

Nurses were divided into two groups based on practical differences in historical training records: the traditional training group (*n* = 126) and the real rescue training group (*n* = 109). Nurses in the traditional training group were defined as those who only participated in simulation scene-based training and theoretical instruction without real rescue training (no signature on the rescue registration book), while nurses in the real rescue training group were defined as those who, in addition to traditional training, also participated in real rescue scenarios. All nurses completed 6 months of standardized training in the Department of Emergency.

### 2.2 Training methods

#### 2.2.1 Traditional training method

Before the training, a training team consisting of the head nurse of the emergency department and two clinical instructors from the same department was formed to develop a training outline guided by the core competencies training curriculum system (12). During the training process, a “teach-first, then practice” approach was adopted, where the instructors taught emergency nursing theory and emergency operational skills, with each session lasting 45 to 60 min, held once a week.

Nurses in the traditional training group received a variety of simulation scenarios ranging from simple to moderately complex based on real emergency rescue situations, such as cardiopulmonary resuscitation (CPR), endotracheal intubation, defibrillation, trauma first aid, and the rescue of critically ill patients. The selected cases were representative, covering all the basic knowledge and skills required for emergency rescue comprehensively. Simulation rescue training was conducted once a week, each session lasting 1 h. Nurses took on roles including doctors, patients, rescue-involved nurses, and family members. After the drills, teaching instructors and resident nurses jointly summarized and reflected on the experience. During the rest of the time, standardized training nurses observed and learned in the actual emergency room, assisted in completing non-critical tasks to help stabilize patients, and became familiar with the department’s workflow and equipment.

Upon completion of the training, standardized training nurses participated in both theoretical and practical examinations (with a maximum score of 100 for each). In addition, patient or family satisfaction surveys were conducted (unified exclusion of critically ill cases), with the overall satisfaction rate calculated as the sum of the satisfied and moderately satisfied rates.

#### 2.2.2 Real rescue training method

Unlike the simulation scenarios of traditional training groups, nurses in the real rescue training group received progressive participation training based on traditional methods. This training was conducted entirely in real emergency resuscitation settings. During the initial phase (months 1–2), standardized training nurses participated as “observers,” focusing on observing the division of labor and collaboration within the resuscitation team, key operational procedures (such as cardiopulmonary resuscitation and airway management), and healthcare communication models (such as SBAR reports). After each resuscitation, they wrote observation notes under the guidance of clinical instructors, summarizing critical response points and teamwork essentials during the resuscitation. In months 3–4, depending on the nurse’s adaptation, their role was adjusted to “assistant participant.” Under the supervision of clinical instructors, they undertook basic resuscitation tasks such as establishing intravenous access, preparing resuscitation medications, and recording vital signs. Instructors provided real-time corrections for any delays or procedural oversights and conducted “one-on-one” feedback sessions within 1 hour after the resuscitation, focusing on response speed (e.g., “Establishing intravenous access took 30 s longer than the standard time; optimize the procedure”) and operational stability under pressure. By months 5–6, nurses with developed capabilities transitioned to the “core participant” role, taking on specific critical tasks within the team (such as assisting with defibrillation, verifying medical orders, and participating in patient condition assessments).

A pilot phase with 10 trainees was conducted to ensure feasibility and refine supervision procedures prior to the main study, though pilot data were not included in the final analysis.

### 2.3 Multidimensional evaluation

Before and after the training, demographic questionnaires, the Triage Decision-Making Inventory (TDMI), Team Climate Inventory (TCI), Maslach Burnout Inventory (MBI), Connor-Davidson Resilience Scale (CD-RISC), and Critical Thinking Disposition Inventory-Chinese Version (CTDI-CV) were distributed. These were completed anonymously, with both the recovery rate and the validity rate being 100%. The primary evaluation indicators were rapid response, team collaboration, and stress resistance. Secondary evaluation indicators included occupational burnout, critical thinking ability, department exit assessment, and nursing satisfaction.

#### 2.3.1 Quick responsiveness ability

The Triage Decision-Making Inventory (TDMI) was used to assess rapid response ability, as it captures cognitive behavior, intuition, and decision-making under time pressure. The TDMI comprises four dimensions: cognitive behavior, experience, intuition, and critical thinking, with a total of 37 items. Each item is rated on a 6-point Likert scale ranging from “strongly disagree” (1 point) to “strongly agree” (6 points). The total score is 222, with higher scores indicating quicker responsiveness abilities. Previous studies report excellent internal consistency (Cronbach’s *α* ≈ 0.95) and good construct validity in healthcare learners. Chinese nursing studies have further confirmed acceptable reliability and stable factorial validity, supporting its use in this population (13).

#### 2.3.2 Teamwork ability

The Team Climate Inventory (TCI) was used to measure team collaboration, focusing on shared vision, interaction, and participatory safety. The TCI includes five subscales: vision, interaction and information sharing, innovation support, task orientation, and participation safety, comprising a total of 38 items. It uses a 7-point Likert scale (1 = “strongly disagree” to 5 = “strongly agree”), with each subscale score calculated as the average of all items in that subscale multiplied by 10 (to convert to a percentage scale). Higher scores indicate a more positive team collaboration atmosphere and stronger collaboration abilities. The Cronbach’s *α* coefficients for the five subscales range from 0.83 to 0.93 (14).

#### 2.3.3 Ability to withstand pressure

The Connor-Davidson Resilience Scale (CD-RISC) was used to evaluate stress resistance, including toughness, strength, and optimism under adversity. The CD-RISC consists of 25 items covering resilience, sense of strength, and optimism. It employs a 5-point Likert scale (0 = “never” to 4 = “always”), with total scores ranging from 0 to 100; higher scores indicate greater psychological resilience. The Cronbach’s *α* coefficient of this scale is 0.89 (15).

#### 2.3.4 Occupational burnout

The Maslach Burnout Inventory (MBI) was included to assess occupational burnout, which is closely linked to nurses’ ability to sustain performance in high-stress environments. The MBI includes three dimensions: emotional exhaustion, depersonalization, and personal accomplishment. The scale consists of 22 items, rated on a 7-point Likert scale (0 = “never” to 6 = “every day”). Higher scores in emotional exhaustion (0–54 points) and depersonalization (0–30 points) indicate stronger occupational burnout, while higher scores in personal accomplishment (0–48 points) indicate weaker occupational burnout. The Cronbach’s *α* coefficient for this scale is 0.86 (16).

#### 2.3.5 Critical thinking ability

The Critical Thinking Disposition Inventory–Chinese Version (CTDI-CV) was used to evaluate critical thinking ability, encompassing truth-seeking, open-mindedness, and systematicity. This scale includes seven dimensions: truth-seeking, open-mindedness, analytical ability, systematicity, confidence in critical thinking, inquisitiveness, and cognitive maturity. Each dimension contains 10 items, rated on a 6-point Likert scale ranging from “strongly disagree” (1 point) to “strongly agree” (6 points). Higher scores indicate a stronger disposition toward critical thinking. The Cronbach’s *α* coefficient for this scale is 0.85 (17).

These instruments were selected because they map directly onto the study’s primary constructs. The TDMI captures decision-making speed and accuracy under triage conditions (rapid response). The TCI reflects perceptions of teamwork and collaboration climate. The CD-RISC evaluates resilience and stress resistance. The MBI provides a countermeasure for stress impact (burnout), while the CTDI-CV assesses critical thinking, which underpins adaptive responses in emergencies.

### 2.4 Ethical approval

This study strictly conforms to the ethical principles outlined in the Declaration of Helsinki and has been reviewed and approved by the Institutional Review Board of the Xijing Hospital. Given that this is a retrospective study with anonymized data, participant-informed consent was waived.

### 2.5 Data analysis

All statistical analyses in this study were performed using SPSS statistical software (version 29.0; developed by SPSS Inc., Chicago, IL, USA). Continuous variables were reported as mean ± standard deviation (M ± SD) based on normality assessed by the Shapiro–Wilk test, while categorical variables were expressed as frequencies and percentages [*n* (%)]. Group differences for continuous variables were analyzed using independent samples *t*-tests, and chi-square tests were used for categorical variables. A two-tailed *p*-value < 0.05 was considered statistically significant.

## 3 Results

### 3.1 Demographics

In Table 1, we present a comparison of the demographics and baseline characteristics between the traditional training group (*n* = 126) and the real rescue training group (*n* = 109). Our analysis revealed no significant differences in age (*p* = 0.397), gender distribution (*p* = 0.594), height (*p* = 0.203), weight (*p* = 0.099), educational background (*p* = 0.932), marital status (*p* = 0.864), living situation (dormitory) (*p* = 0.980), work experience before joining (*p* = 0.827), pre-job theoretical score (*p* = 0.102), skill operation score (*p* = 0.201), as well as prior rotation experience in ICU (*p* = 0.879) and CCU (*p* = 0.855) between the two groups. All *p* values were above the significance level of 0.05, indicating that the two groups were comparable in terms of these baseline parameters.

**Table 1:** Comparison of baseline characteristics between the traditional training and the real rescue training groups.

Parameter	Traditional group (*n* = 126)	Real rescue group (*n* = 109)	*t*/*χ*^2^	*p* value
Age (years)	24.32 ± 1.46	24.17 ± 1.38	0.849	0.397
Gender, *n* (%)			0.284	0.594
Male	18 (14.3)	13 (11.9)		
Female	108 (85.7)	96 (88.1)		
Height (cm)	166.28 ± 3.51	165.73 ± 2.93	1.276	0.203
Weight (kg)	56.26 ± 5.63	57.31 ± 4.06	1.657	0.099
Educational background, *n* (%)			0.007	0.932
Bachelor’s degree or above	100 (79.4)	87 (79.8)		
Junior college	26 (20.6)	22 (20.2)		
Marital status, *n* (%)			0.029	0.864
Married	13 (10.3)	12 (11.0)		
Single	113 (89.7)	97 (89.0)		
Living situation (dormitory), *n* (%)			0.001	0.980
Yes	112 (88.9)	97 (89.0)		
No	14 (11.1)	12 (11.0)		
Work experience, *n* (%)			0.048	0.827
Yes	15 (11.9)	14 (12.8)		
No	111 (88.1)	95 (87.2)		
Pre-job training score (points)				
Theoretical score	91.93 ± 2.47	92.45 ± 2.34	1.644	0.102
Skill operation score	92.36 ± 1.79	92.08 ± 1.51	1.284	0.201
Prior rotation experience, *n* (%)				
ICU	37 (29.4)	33 (30.3)	0.023	0.879
CCU	29 (23.0)	24 (22.0)	0.033	0.855

### 3.2 Quick responsiveness ability

When comparing the Triage Decision-Making Inventory (TDMI) scores between the Traditional training group and the Real rescue training group, no significant differences were observed in cognitive behavior, experience sharing, intuition, and critical thinking before training (all *p* > 0.05) (Table 2). However, after completing their respective training programs, all assessed parameters showed significant improvements within each group (all *p* < 0.05). For cognitive behavior, the Real rescue training group showed a significant increase compared to the Traditional training group (*p* = 0.009). Similarly, in experience sharing, the Real rescue training group exhibited higher scores than the Traditional training group (*p* = 0.01). Regarding intuition, the Real rescue training group scored significantly higher than the Traditional training group (*p* = 0.009). For critical thinking, the Real rescue training group also demonstrated significantly higher scores compared to the Traditional training group (*p* = 0.001). In summary, while both training methods led to significant improvements in participants’ triage decision-making skills, the Real rescue training group outperformed the Traditional training group in every measured aspect post-training. This suggests that the Real rescue training approach may be more effective in enhancing specific skills related to quick responsiveness ability in triage settings.

**Table 2:** Comparison of TDMI scores between traditional and real rescue training groups (points, mean ± SD).

Parameter	Timepoint	Traditional group (*n* = 126)	Real rescue group (*n* = 109)	*t*	*p* value
Cognitive behavior	Before training	21.52 ± 2.13	21.38 ± 2.07	0.533	0.595
	After training	35.76 ± 3.25ᵃ	36.84 ± 2.97ᵃ	2.623	0.009
Experience sharing	Before training	19.87 ± 1.92	19.95 ± 1.88	0.324	0.746
	After training	32.85 ± 2.78ᵃ	33.72 ± 2.51ᵃ	2.507	0.013
Intuition	Before training	22.63 ± 2.34	22.95 ± 3.38	0.813	0.417
	After training	38.65 ± 3.42ᵃ	39.72 ± 2.85ᵃ	2.619	0.009
Critical thinking	Before training	22.62 ± 2.05	22.14 ± 2.11	1.754	0.081
	After training	41.48 ± 3.16ᵃ	42.81 ± 2.94ᵃ	3.314	0.001

Values are presented as mean ± standard deviation. *p*-values were obtained using independent-samples *t*-test.

TDMI, Triage Decision-Making Inventory.

ᵃ *p* < 0.05 compared with the same group before training.

### 3.3 Teamwork ability

In comparing the Team Climate Inventory (TCI) scores between the Traditional training group and the Real rescue training group, no significant differences were observed in any parameters before training, including vision, interaction, and information sharing, support for innovation, task orientation, and participation safety (all *p* > 0.05) (Table 3). After completing their respective training programs, all assessed parameters showed significant improvements within each group compared to their pre-training scores (all *p* < 0.05). Post-training comparisons revealed that the Real rescue training group consistently scored higher than the Traditional training group across all evaluated parameters. Specifically, the Real rescue training group demonstrated better performance in vision (*p* = 0.02). Similarly, the Real rescue training group outperformed the Traditional training group in interaction and information sharing (*p* = 0.006). The Real rescue training group also exhibited higher scores in support for innovation (*p* = 0.01). Furthermore, in task orientation, the Real rescue training group achieved higher scores (*p* = 0.007). Lastly, the Real rescue training group showed superior results in participation safety (*p* = 0.006). These findings suggest that while both training methods significantly enhanced participants’ teamwork abilities, the Real rescue training approach may be more effective in fostering a positive team climate and enhancing specific teamwork skills.

**Table 3:** Comparison of TCI scores between traditional and real rescue training groups (points, mean ± SD).

Parameter	Timepoint	Traditional group (*n* = 126)	Real rescue group (*n* = 109)	*t*	*p* value
Vision	Before training	32.15 ± 4.27	32.08 ± 4.31	0.117	0.907
	After training	38.82 ± 3.95ᵃ	39.87 ± 3.12ᵃ	2.294	0.023
Interaction & information sharing	Before training	34.62 ± 3.85	34.73 ± 3.91	0.211	0.833
	After training	42.05 ± 3.62ᵃ	43.25 ± 2.98ᵃ	2.787	0.006
Support for innovation	Before training	29.87 ± 3.42	29.95 ± 3.38	0.186	0.853
	After training	37.74 ± 3.28ᵃ	38.72 ± 2.85ᵃ	2.442	0.015
Task orientation	Before training	36.41 ± 4.13	36.35 ± 4.05	0.119	0.906
	After training	43.93 ± 3.75ᵃ	45.12 ± 2.93ᵃ	2.741	0.007
Participation safety	Before training	31.28 ± 3.67	31.33 ± 3.72	0.110	0.912
	After training	40.15 ± 3.41ᵃ	41.33 ± 3.05ᵃ	2.788	0.006

Values are presented as mean ± standard deviation. *p*-values were obtained using an independent-samples *t*-test.

TCI, Team Climate Inventory.

ᵃ *p* < 0.05 compared with the same group before training.

### 3.4 Ability to withstand pressure

**Figure 1:**
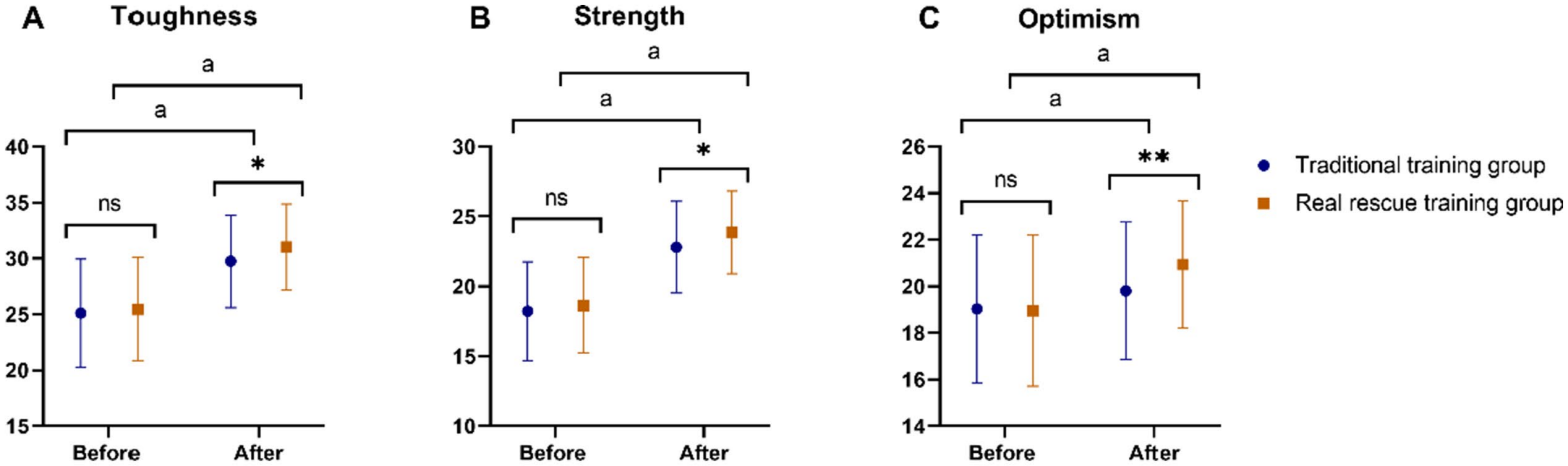
Comparison of the CD-RISC score between the two groups before and after training. **(A)** Toughness; **(B)** Strength; **(C)** Optimism; CD-RISC, Connor-Davidson Resilience Scale. Ns, No significant difference; *: *p* < 0.05; **: *p* < 0.01; ^a^: *p* < 0.05, compared to the same group before training.

In the comparison of Connor-Davidson Resilience Scale (CD-RISC) scores between the Traditional training group and the Real rescue training group, no significant differences were observed in any parameters before training: toughness (*p* = 0.571), strength (*p* = 0.348), and optimism (*p* = 0.879) (Figure 1). After completing their respective training programs, all assessed parameters showed significant improvements within each group compared to their pre-training scores (all *p* < 0.05). Post-training comparisons revealed that the Real rescue training group consistently scored higher than the Traditional training group across all evaluated parameters. Specifically, for toughness, the Real rescue training group demonstrated better resilience with a lower *p* value indicating statistical significance (*p* = 0.01). Similarly, in strength, the Real rescue training group outperformed the Traditional training group (*p* = 0.01), showing greater improvement in this aspect. Furthermore, for optimism, the Real rescue training group also achieved higher scores compared to the Traditional training group (*p* = 0.003), reflecting more positive outlooks and attitudes. These findings suggest that while both training methods significantly enhanced participants’ resilience as measured by the CD-RISC, the Real rescue training group exhibited superior outcomes in all assessed dimensions of resilience post-training.

### 3.5 Occupational burnout

In assessing the Maslach Burnout Inventory (MBI) scores between the Traditional training group and the Real rescue training group, initial comparisons indicated no significant differences in any parameters before training: emotional exhaustion (EE) (*p* = 0.903), depersonalization (DP) (*p* = 0.881), and personal accomplishment (PA) (*p* = 0.940) (Table 4). Following the completion of their respective training programs, all parameters demonstrated notable improvements within each group relative to pre-training levels (all *p* < 0.05). Post-training evaluations revealed that the Real rescue training group exhibited more favorable outcomes compared to the Traditional training group across all measured aspects. Specifically, regarding emotional exhaustion, the Real rescue training group showed a greater reduction (*p* = 0.008), indicating lower levels of burnout. Similarly, for depersonalization, participants in the Real rescue training group reported significantly lower scores (*p* = 0.001), reflecting diminished feelings of detachment or cynicism. In addition, in terms of personal accomplishment, the Real rescue training group achieved higher scores (*p* = 0.008), suggesting enhanced perceptions of efficacy and achievement. These results indicate that while both training methods contributed to reducing burnout and enhancing personal accomplishment, the Real Rescue training approach was particularly effective in achieving these outcomes (Table 5).

**Table 4:** Comparison of MBI scores between traditional and real rescue training groups (points, mean ± SD).

Parameter	Timepoint	Traditional group (*n* = 126)	Real rescue group (*n* = 109)	*t*	*p* value
Emotional exhaustion (EE)	Before training	28.73 ± 5.42	28.65 ± 5.37	0.122	0.903
	After training	24.25 ± 5.83ᵃ	22.41 ± 4.72ᵃ	2.665	0.008
Depersonalization (DP)	Before training	12.85 ± 3.16	12.78 ± 3.21	0.150	0.881
	After training	11.21 ± 3.45ᵃ	9.85 ± 2.93ᵃ	3.216	0.001
Personal accomplishment (PA)	Before training	32.13 ± 4.25	32.17 ± 4.32	0.075	0.940
	After training	38.25 ± 4.52ᵃ	39.72 ± 3.85ᵃ	2.658	0.008

ᵃ *p* < 0.05 compared with the same group before training.

**Table 5:** Comparison of CTDI-CV score between two groups (points, M ± SD).

Parameter	Timepoint	Traditional group (*n* = 126)	Real rescue group (*n* = 109)	*t*	*p* value
Truth seeking	Before training	36.93 ± 3.07	37.13 ± 3.22	0.478	0.633
	After training	38.17 ± 3.53ᵃ	39.19 ± 3.82ᵃ	2.141	0.033
Open-mindedness	Before training	36.21 ± 3.04	36.63 ± 3.32	1.015	0.311
	After training	38.23 ± 3.32ᵃ	39.31 ± 3.25ᵃ	2.522	0.012
Analyticity	Before training	35.93 ± 2.72	36.44 ± 2.83	1.403	0.162
	After training	38.02 ± 3.13ᵃ	39.05 ± 3.72ᵃ	2.290	0.023
Systematicity	Before training	37.97 ± 3.07	38.31 ± 3.06	0.851	0.396
	After training	38.96 ± 3.16ᵃ	40.02 ± 3.09ᵃ	2.589	0.010
Critical thinking self-confidence	Before training	35.97 ± 3.05	36.13 ± 3.26	0.404	0.687
	After training	39.17 ± 3.29ᵃ	40.18 ± 3.42ᵃ	2.308	0.022
Inquisitiveness	Before training	36.93 ± 3.27	37.22 ± 3.42	0.653	0.514
	After training	39.33 ± 2.96ᵃ	40.25 ± 3.17ᵃ	2.298	0.022
Cognitive maturity	Before training	38.06 ± 2.38	38.55 ± 3.52	1.228	0.221
	After training	40.07 ± 3.24ᵃ	41.25 ± 3.23ᵃ	2.795	0.006

Values are presented as mean ± standard deviation. *p*-values were obtained using an independent-samples *t*-test.

CTDI-CV, Critical Thinking Disposition Inventory–Chinese Version.

ᵃ *p* < 0.05 compared with the same group before training.

### 3.6 Critical thinking ability

In the comparison of Critical Thinking Disposition Inventory-Chinese Version (CTDI-CV) scores between the Traditional training group and the Real rescue training group, no significant differences were observed in any parameters before training, including truth seeking, open-mindedness, analyticity, systematicity, critical thinking self-confidence, inquisitiveness, and cognitive maturity (all *p* > 0.05). After completing their respective training programs, all assessed parameters showed significant improvements within each group compared to their pre-training scores (all *p* < 0.05). Post-training comparisons revealed that the Real rescue training group consistently scored higher than the Traditional training group across all evaluated parameters. Specifically, for truth seeking, the Real rescue training group demonstrated better performance (*p* = 0.03). Similarly, in open-mindedness, the Real rescue training group outperformed the Traditional training group (*p* = 0.01). The Real rescue training group also exhibited higher scores in analyticity (*p* = 0.02). For systematicity, the Real rescue training group achieved higher scores (*p* = 0.010). In terms of critical thinking self-confidence, the Real rescue training group showed superior results (*p* = 0.02). In addition, for inquisitiveness, the Real rescue training group also performed better (*p* = 0.02). Lastly, the Real rescue training group showed higher scores in cognitive maturity (*p* = 0.006). These findings suggest that while both training methods significantly improved participants’ critical thinking dispositions, the Real rescue training approach was more effective in fostering these skills.

### 3.7 Department exit assessment

**Figure 2:**
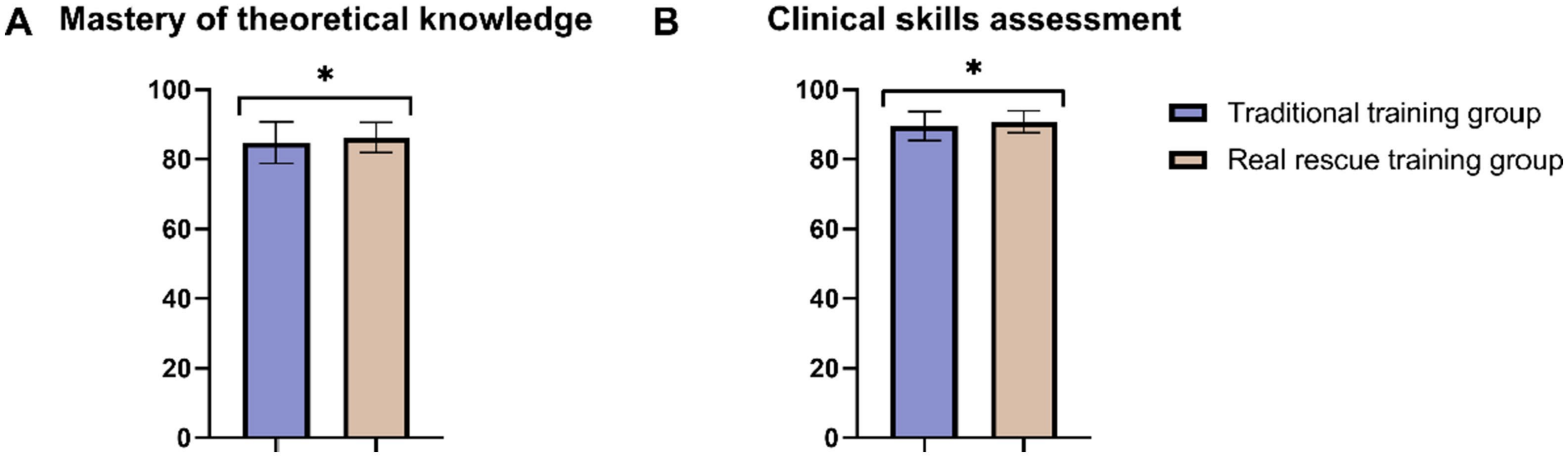
Comparison of department exit assessment between two groups. **(A)** Mastery of theoretical knowledge; **(B)** Clinical skills assessment. *: *p* < 0.05.

In the comparison of department exit assessment scores between the Traditional training group and the Real rescue training group, significant differences were observed in both evaluated parameters post-training (Figure 2). The Real rescue training group demonstrated higher mastery of theoretical knowledge compared to the Traditional training group (*p* = 0.02), indicating a stronger grasp of essential concepts. In addition, in the clinical skills assessment, the Real rescue training group also outperformed the Traditional training group (*p* = 0.01), showcasing superior practical abilities. These results highlight that while both training methods are effective, the Real rescue training approach appears to be more efficacious in enhancing both theoretical knowledge and practical skills.

### 3.8 Nursing satisfaction

In evaluating the nursing satisfaction between the Traditional training group and the Real rescue training group, significant differences were observed in the overall satisfaction rates (Table 6). The Real rescue training group reported a higher overall satisfaction rate compared to the Traditional training group (*p* = 0.01). Specifically, while the proportion of nurses who were satisfied was slightly higher in the Real rescue training group, the most notable difference was in the dissatisfied category, where only 0.92% of nurses in the Real rescue training group expressed dissatisfaction compared to 7.94% in the Traditional training group. The proportion of nurses reporting moderate satisfaction was similar between the two groups. These findings indicate that the Real rescue training approach not only achieves a higher overall satisfaction rate but also significantly reduces the proportion of dissatisfied nurses.

**Table 6:** Comparison of nursing satisfaction between two groups [*n* (%)].

Parameter	Traditional training group (*n* = 126)	Real rescue training group (*n* = 109)	*χ* ^2^	*p*
Overall satisfaction rate	116 (92.06%)	108 (99.08%)	6.453	0.011
Satisfied	86 (68.25%)	81 (74.31%)		
Moderately satisfied	30 (23.81%)	27 (24.77%)		
Dissatisfied	10 (7.94%)	1 (0.92%)		

## 4 Discussion

This study investigated whether a structured program of progressive participation in real emergency resuscitations embedded alongside standard simulation and classroom teaching improves core competencies in newly hired nurses compared with traditional simulation-only training. Across validated instruments, the real rescue group showed greater post-training gains in rapid decision-making (TDMI), team climate (TCI), resilience (CD-RISC), burnout (MBI), and critical-thinking dispositions (CTDI-CV), as well as superior performance on department exit assessments and higher training satisfaction.

Real rescue participation yielded broader and larger improvements than traditional training alone. The pattern of effects suggests complementary mechanisms. First, experiential learning in authentic, time-pressured contexts likely accelerated schema formation and application, consistent with the theory that knowledge is consolidated through concrete experience and reflective observation (18, 19). Second, the social learning inherent in real teams observing experts, rehearsing roles, and receiving immediate interprofessional feedback appears to strengthen communication, role clarity, and psychological safety, aligning with your TCI improvements (20, 21). Third, stress inoculation via graded exposure → observer → assistant → supervised participant likely enhanced coping and self-efficacy, explaining the resilience gains and burnout reductions (22–24).

Beyond these established mechanisms, two additional factors may help explain the breadth of benefits observed. First, elements of cognitive-load optimization, briefing, role priming, and focused task assignment may have reduced extraneous load and preserved working memory for clinical reasoning, which is consistent with the stronger gains in TDMI and CTDI-CV. Second, the “consequential validity” of real cases (i.e., decisions with real patient impact) can heighten attentional engagement and memory encoding, plausibly contributing to the meaningful improvements you observed across instruments.

The results are directionally consistent with work showing that high-fidelity and scenario-based learning improves clinical decision-making, teamwork, and adaptability under pressure, while authentic exposure can potentiate stress resilience (22–28). The present study extends that evidence by demonstrating advantages of supervised real-case participation over simulation alone across multiple validated domains concurrently, including critical-thinking dispositions (29, 30) and practical exit assessments (31). This concurrent, cross-construct improvement strengthens the case that authentic clinical participation adds value beyond simulation-based mastery of protocols.

Findings support embedding a phased “observe-assist-perform under supervision” pathway into orientation for emergency nurses. Programs should formalize instructor-to-trainee ratios, pre-brief/debrief structures, and real-time coaching checklists to safeguard patient safety and learner well-being. Where real-case opportunities are limited, hybrid models (simulation priming followed by targeted real-case participation) may achieve similar competency gains while maintaining safety and feasibility. Given the reductions in depersonalization and emotional exhaustion, linking real rescue curricula with wellness resources (peer debriefs, resilience micro-skills) may further buffer burnout. Hospitals planning scale-up should consider implementation supports (scheduling, case selection criteria, competency sign-offs, and documentation standards) to ensure fidelity.

### 4.1 Strengths and limitations

Strengths include the use of multiple validated instruments spanning decision-making, teamwork, resilience/burnout, and critical thinking; a graded participation design aligned with experiential and stress-inoculation principles; and convergent improvements in both psychometric scores and exit-assessment performance. Important limitations remain. This was a single-center, non-randomized study with assessments immediately post-training, limiting causal inference and durability claims. Instruments were self-report (albeit validated), which may not fully capture real-time performance. Unmeasured confounding (e.g., informal mentorship, prior informal exposure) could contribute to effects. Potential Hawthorne and expectancy effects heightened motivation in the real rescue cohort, cannot be excluded. Finally, resource and ethical safeguards (supervision capacity, patient consent processes, escalation thresholds) may affect generalizability across settings.

### 4.2 Future directions

Multi-center randomized or stepped-wedge trials are needed to test causal effects and estimate the durability of gains at 3–12 months. Objective performance endpoints (time-to-intervention, protocol adherence, error rates in simulated code events, team communication metrics) should complement self-report. Dose–response evaluations (number/intensity of real cases) can guide efficient program design. Implementation studies should examine feasibility, staffing, and cost-effectiveness, and equity/ethics frameworks (psychological safety, patient consent) should be prospectively embedded. Comparative trials of hybrid models (simulation→targeted real cases) versus simulation-only can clarify the incremental value of authentic exposure while maintaining safety.

As for application, the emergency department’s rescue training approach may be effectively scaled to other acute-care settings, including intensive care units, coronary care units, and trauma services, where swift response, collaboration, and resilience are paramount. To guarantee safe and successful implementation, training must adhere to a systematic progression, initiating with observation, progressing to assistance roles, and concluding with supervised active involvement. Supportive supervision and planned debriefings are equally crucial, as they alleviate psychological strain and enhance reflective learning. Institutional adaptation may necessitate customization based on available resources, patient demographics, and staff-to-patient ratios; nonetheless, the fundamental premise of integrating genuine, supervised clinical experience into standardized training might enhance nurse readiness in many high-stakes settings.

## 5 Conclusion

This study demonstrates that a structured program of progressive participation in real emergency resuscitations, integrated with traditional simulation and didactic teaching, is highly effective for emergency nurse standardized training. Compared to traditional simulation-based training alone, this approach improves rapid response capability, team collaboration, stress resistance, and critical thinking more significantly. It also results in lower occupational burnout, higher assessment scores, and substantially greater job satisfaction among trainees. The phased model, progressing from Observer to Assistant Participant to Core Participant with close supervision and dedicated debriefing, is essential for maximizing learning while managing risks. The compelling advantages advocate for the integration of meticulously designed real rescue experiences as a fundamental element of emergency nursing education, enhancing nurses’ preparedness for the realities of clinical practice. Future multi-center randomized trials with long-term outcome assessment and incorporation of objective performance measures would further strengthen the evidence base.

## Data Availability

The original contributions presented in the study are included in the article/supplementary material, further inquiries can be directed to the corresponding author.

## References

[1] Drennan J Murphy A Mccarthy VJC Ball J Duffield C Crouch R. The association between nurse staffing and quality of care in emergency departments: a systematic review. Int J Nurs Stud. (2024) 153:104706. doi: 10.1016/j.ijnurstu.2024.104706, PMID: 38447488

[2] Pearce S Marr E Shannon T Marchand T Lang E. Overcrowding in emergency departments: an overview of reviews describing global solutions and their outcomes. Intern Emerg Med. (2024) 19:483–91. doi: 10.1007/s11739-023-03477-4, PMID: 38041766

[3] Elendu C Amaechi DC Okatta AU Amaechi EC Elendu TC Ezeh CP. The impact of simulation-based training in medical education: a review. Medicine. (2024) 103:e38813. doi: 10.1097/MD.0000000000038813, PMID: 38968472 PMC11224887

[4] Pietersen PI Bjerrum F Tolsgaard MG Konge L Andersen SAW. Standard setting in simulation-based training of surgical procedures: a systematic review. Ann Surg. (2022) 275:872–82. doi: 10.1097/SLA.0000000000005209, PMID: 34520423

[5] Alyateem S Al-Ruzzieh M Shtayeh B Alloubani A. Comparing the efficacy of single-skill and multiple-skill simulation scenarios in advancing clinical nursing competency. Heliyon. (2024) 10:e29931. doi: 10.1016/j.heliyon.2024.e29931, PMID: 38720750 PMC11076845

[6] Kiessling A Amiri C Arhammar J Lundbäck M Wallingstam C Wikner J. Interprofessional simulation-based team-training and self-efficacy in emergency medicine situations. J Interprof Care. (2022) 36:873–81. doi: 10.1080/13561820.2022.2038103, PMID: 35341425

[7] Dawood E Alshutwi SS Alshareif S Shereda HA. Evaluation of the effectiveness of standardized patient simulation as a teaching method in psychiatric and mental health nursing. Nurs Rep. (2024) 14:1424–38. doi: 10.3390/nursrep14020107, PMID: 38921717 PMC11206419

[8] Bandura A Walters RH. Social learning theory. Englewood Cliffs, NJ: Prentice Hall (1977).

[9] Meshel A Iavicoli L Dilos B Agriantonis G Kessler S Fairweather P. Strategic educational expansion of trauma simulation initiative via a plan-do-study-act ramp. West J Emerg Med. (2023) 24:76–8. doi: 10.5811/westjem.2022.12.57735, PMID: 36735012 PMC9897251

[10] Wallander Karlsen MM Sørensen AL Finsand C Sjøberg M Lieungh M Stafseth SK. Combining clinical practice and education in critical care nursing-a trainee program for registered nurses. Nurs Open. (2023) 10:3666–76. doi: 10.1002/nop2.1617, PMID: 36709494 PMC10170886

[11] Eickelmann AK Waldner NJ Huwendiek S. Teaching the technical performance of bronchoscopy to residents in a step-wise simulated approach: factors supporting learning and impacts on clinical work - a qualitative analysis. BMC Med Educ. (2021) 21:597. doi: 10.1186/s12909-021-03027-6, PMID: 34856967 PMC8641234

[12] Xie L Feng M Cheng J Huang S. Developing a core competency training curriculum system for emergency trauma nurses in China: a modified Delphi method study. BMJ Open. (2023) 13:e066540. doi: 10.1136/bmjopen-2022-066540, PMID: 37130690 PMC10163488

[13] Smith A Kj C. Triage decision-making skills: a necessity for all nurses. J Nurses Staff Dev. (2010) 26:E14–9. doi: 10.1097/NND.0b013e3181bec1e6 20098163

[14] Ouwens M Hulscher M Akkermans R Hermens R Grol R Wollersheim H. The team climate inventory: application in hospital teams and methodological considerations. Qual Saf Health Care. (2008) 17:275–80. doi: 10.1136/qshc.2006.021543, PMID: 18678725

[15] Connor K Davidson J. Development of a new resilience scale: the Connor‐Davidson resilience scale (CD‐RISC). Depress Anxiety. (2003) 18:76–82. doi: 10.1002/da.10113 12964174

[16] Coker AC Omoluabi PF. Validation of Maslach burnout inventory. Ife Psychol. (2009) 17:231–42. doi: 10.4314/ifep.v17i1.43750

[17] Hwang SY Yen M Lee BO Huang MC Tseng HF. A critical thinking disposition scale for nurses: short form. J Clin Nurs. (2010) 19:3171–6. doi: 10.1111/j.1365-2702.2010.03343.x, PMID: 21040020

[18] Lowe AE Kraft C Kortepeter MG Hansen KF Sanger K Johnson A. Developing a rapid response single Irb model for conducting research during a public health emergency. Health Sec. (2022) 20:S-60–70. doi: 10.1089/hs.2021.0181, PMID: 35544310

[19] Senken B Welch J Sarmiento E Weinstein E Cushman E Kelker H. Factors influencing emergency medicine worker shift satisfaction: a rapid assessment of wellness in the emergency department. JACEP Open. (2024) 5:e13315. doi: 10.1002/emp2.13315, PMID: 39479347 PMC11521350

[20] Dawe J Cronshaw H Frerk C. Learning from the multidisciplinary team: advancing patient care through collaboration. Br J Hosp Med. (2024) 85:1–4. doi: 10.12968/hmed.2023.0387, PMID: 38815972

[21] Hosseini M Heydari A Reihani H Kareshki H. Resuscitation team members 'experiences of teamwork: a qualitative study. Iran J Nurs Midwifery Res. (2022) 27:439–45. doi: 10.4103/ijnmr.ijnmr_294_21, PMID: 36524141 PMC9745842

[22] Lapierre A Lavoie P Castonguay V Lonergan AM Arbour C. The influence of the simulation environment on teamwork and cognitive load in novice trauma professionals at the emergency department: piloting a randomized controlled trial. Int Emerg Nurs. (2023) 67:101261. doi: 10.1016/j.ienj.2022.101261, PMID: 36804137

[23] Joseph A Jose TP. Coping with distress and building resilience among emergency nurses: a systematic review of mindfulness-based interventions. Indian J Crit Care Med. (2024) 28:785–91. doi: 10.5005/jp-journals-10071-24761, PMID: 39239180 PMC11372665

[24] Lan L Zhou M Wang L Chen X Dai M Zhang J. Enhancing emergency nurses' disaster nursing ability and psychological resilience: a randomized controlled trial. Emerg Med Int. (2023) 2023:1–9. doi: 10.1155/2023/6108057, PMID: 38054165 PMC10695688

[25] Wang Y Liang Y Zheng X Zhang X Feng L. Exploring the genuine psychological experiences of novice nurses at emergency resuscitation events: a qualitative interview study. Heliyon. (2024) 10:e35153. doi: 10.1016/j.heliyon.2024.e35153, PMID: 39157395 PMC11328028

[26] Kavakli O Konukbay D. How simulation training for nursing students in emergency internships affects triage decision-making and anxiety: a quasi-experimental study. Heliyon. (2024) 10:e35626. doi: 10.1016/j.heliyon.2024.e35626, PMID: 39170198 PMC11336885

[27] Murphy M Mccloughen A Curtis K. Using theories of behaviour change to transition multidisciplinary trauma team training from the training environment to clinical practice. Implement Sci. (2019) 14:43. doi: 10.1186/s13012-019-0890-6, PMID: 31036023 PMC6489197

[28] Arnal-Velasco D Heras-Hernando V. Learning from errors and resilience. Curr Opin Anaesthesiol. (2023) 36:376–81. doi: 10.1097/ACO.0000000000001257, PMID: 36794873

[29] Choi MJ Kim KJ. Effects of team-based mixed reality simulation program in emergency situations. PLoS One. (2024) 19:e0299832. doi: 10.1371/journal.pone.0299832, PMID: 38422080 PMC10903827

[30] Blissett S Skinner J Banner H Cristancho S Taylor T. How do residents respond to uncertainty with peers and supervisors in multidisciplinary teams? Insights from simulations with epistemic fidelity. Adv Simul. (2024) 9:8. doi: 10.1186/s41077-024-00281-8, PMID: 38347654 PMC10863229

[31] Panda S Dash M John J Rath K Debata A Swain D. Challenges faced by student nurses and midwives in clinical learning environment - a systematic review and meta-synthesis. Nurse Educ Today. (2021) 101:104875. doi: 10.1016/j.nedt.2021.104875, PMID: 33774528

